# Assessing Food Safety Practices and Knowledge of Consumer Health Risks Among Street Food Vendors in Rajshahi City, Bangladesh

**DOI:** 10.7759/cureus.100244

**Published:** 2025-12-28

**Authors:** Nazia Tabassum, Faiyaz A Khan, Rashik Shadman, Fahad Mahmood, Md. Foyzur Rahman, Rowshan Jahan

**Affiliations:** 1 Department of Occupational and Environmental Health, National Institute of Preventive and Social Medicine (NIPSOM), Dhaka, BGD; 2 Medicine and Surgery, Sarkari Karmachari Hospital, Dhaka, BGD; 3 Department of Community Medicine, National Institute of Preventive and Social Medicine (NIPSOM), Dhaka, BGD; 4 Department of Nutrition and Biochemistry, National Institute of Preventive and Social Medicine (NIPSOM), Dhaka, BGD

**Keywords:** food safety practices, health risk, hygiene, rajshahi city, street food vendors

## Abstract

Background: Street food (SF) plays a vital role in food security in urban and busy areas. However, inadequate hygiene practices and limited awareness of foodborne risks among vendors create significant public health concerns. This cross-sectional study was conducted among SF vendors in Rajshahi City, Bangladesh, and aimed to assess the food safety practices and health risk knowledge among SF vendors.

Methods: A cross-sectional study was conducted from July to December 2023, involving 350 SF vendors selected using systematic random sampling. Data were collected through face-to-face interviews using a semi-structured questionnaire and analyzed with IBM SPSS Statistics version 27. Chi-square test or Fisher’s exact test was employed to identify associations, with a significance threshold set at p < 0.05.

Results: Majority of the vendors were male (78.6%), aged 21-40 years, and shop owners (74.6%). While 72.3% reported always washing hands before food processing and 71.7% after toilet use, however, for hygiene practices, none of the vendors reported using gloves, caps, or masks, and only 2.3% reported wearing aprons. Unsafe behaviors, such as handling food while ill (9.7%) and not sanitizing utensils (31.7%), were prevalent. Awareness of foodborne illnesses was mixed; although 63.4% identified food poisoning as a risk, knowledge of pathogens like *Salmonella* and *Staphylococcus* was low. Significant associations (p < 0.05) were found between hygiene practices and factors such as experience, employment status, personal habits, and water source. Similarly, knowledge levels were associated with gender, education, prior experience, income, and employment status of the vendors.

Conclusion: The study highlights critical deficiencies in food safety practices and health risk awareness among SF vendors in Rajshahi City. These findings underscore the urgent need for targeted training programs, vendor education, regulatory enforcement, and health education interventions to mitigate foodborne disease risks in informal food sectors.

## Introduction

The rising urban population led to a surge in informal businesses, such as street food (SF) vendors [1]. SF and beverages are defined as ready-to-eat items prepared and sold by vendors in public locations, particularly on streets and in busy areas, such as bus and train stations in Bangladesh and many other countries worldwide [1,2]. SF mirrors the dietary practices of major urban centers, especially in the Far East, where it has become an integral part of the local lifestyle. Globally, it is estimated that around 2.5 billion people consume SF each day. This trend is especially evident in developing countries, where a substantial share of household expenditure is dedicated to eating outside the home. For instance, in Latin America, nearly 30% of consumer budgets are spent on SF [3-5].

SF represents an important aspect of national culinary heritage. It fosters the spread of local eating customs on a global scale and helps safeguard cultural and social traditions embedded in local cuisine. In addition, SF serves as a tourist attraction, offering authentic culinary experiences that enhance a nation’s tourism sector. For vendors, it is also a vital source of income, contributing significantly to employment. The popularity of SF stems from its flavorful taste, affordability, accessibility, cultural relevance, social importance, and nutritional benefits [6].

Despite its popularity, food safety remains a pressing issue in many developing nations, especially in China, where SF holds a prominent place in the culinary landscape. Unsafe food handling techniques and unsanitary environments, along with limited regulatory control and guidance, have contributed to the spread of foodborne illnesses, high levels of coliform bacteria, and antibiotic-resistant infections. Thus, ensuring the safety of SF is critical for building a robust and healthy food system. In Southeast Asia, Bangladesh is particularly well known for its wide variety and affordable SF, typically sold in busy public areas [7]. Around 130 distinct SF items are available and are consumed by approximately 2.5 million people each day [8]. However, despite their popularity and perceived nutritional value, many of these foods do not meet safety standards [9]. The lack of awareness and weaknesses in the existing food safety regulations contribute to the country's poor food safety record [10]. To address these challenges, the Food and Agriculture Organization of the United Nations suggests implementing HACCP (Hazard Analysis and Critical Control Points) principles or ensuring the presence of competent food safety authorities, particularly in high-density areas [11].

Therefore, the aim of this study was to assess food safety practices and the level of health risk knowledge among SF vendors in Rajshahi City, Bangladesh. The findings of this research may support public health authorities and urban planners in formulating targeted interventions to improve food hygiene behaviors and reduce foodborne illnesses in the city. We hypothesized that higher education, longer experience, and access to supply water would be associated with better food safety practices and higher health-risk knowledge.

## Materials and methods

Study design and area

The cross-sectional study was conducted from 1 July 2023 to 31 December 2023 in Rajshahi City, a major urban area in north-western Bangladesh with a high density of SF vendors. Data were collected from multiple wards, including markets, transport hubs, and areas near educational institutions.

Source of population

The source population comprised all SF vendors operating in various locations throughout Rajshahi City during the study period. The study population included those SF vendors who were actively engaged in food preparation and selling and who were selected for participation using a systematic random sampling technique based on their accessibility and willingness to respond.

Inclusion and exclusion criteria

SF vendors who had been engaged in food preparation and selling in Rajshahi City for at least six months prior to the data collection period were included in the study. Vendors who were absent or not actively operating during the time of data collection were excluded.

Sample size determination

The sample size was calculated using the single population proportion formula: n = [(Z ) ² × p × (1 − p)] / d², where Z is the critical value for a 95% confidence interval (1.96), p is the assumed prevalence of good food safety practices (taken as 50% due to lack of prior data), and d is the margin of error (5%). Substituting the values yields: n = [(1.96) ² × 0.5 × 0.5] / (0.05) ² = 384. Due to logistical and resource limitations, a final sample of 350 respondents was selected. Participants were proportionally allocated from various vending areas across Rajshahi City. This adjustment still ensures reasonable statistical power and representation of the target population.

Proportional allocation of the sample and sampling procedures

Prior to sample selection, a preliminary ward-wise field survey was conducted to develop a complete sampling frame. This survey identified 700 active SF vendors across all wards of Rajshahi City Corporation who were engaged in food preparation and sale at the time of data collection. Each vendor was assigned a unique identification number based on ward number and sequential order of listing, along with documentation of stall location and nearby permanent landmarks to ensure traceability.

The final sample size (n = 350) was proportionally allocated to each ward based on the number of vendors listed in that ward relative to the total sampling frame. The number of vendors selected from each ward was calculated using the formula:

ni = (Ni / N) × n,

where ni is the sample size for ward i, Ni is the total number of vendors in ward i, N is the total number of vendors in all wards (700), and n is the final sample size (350).

Within each ward, vendors were selected using systematic random sampling. The sampling interval (k) was calculated by dividing the total number of listed vendors in the ward (Ni) by the allocated sample size for that ward (ni). A random starting point between 1 and k was chosen using a random number table, after which every kth vendor on the list was selected until the required sample size for that ward was achieved.

If a selected vendor was unavailable at the time of visit, at least one revisit was attempted. If the vendor remained unavailable or declined participation, the next vendor on the list was selected to maintain the sampling interval. Only vendors who provided informed consent were included in the final analysis.

Food safety knowledge and practices questionnaire

A structured, interviewer-administered questionnaire was used to collect information on sociodemographic characteristics, food safety practices, and food safety knowledge among the SF vendors. The data collection questionnaire was taken from previously published and validated instruments assessing food safety knowledge and practices among SF vendors [9,12]. The questionnaire items and response options were retained in their original structure. The tool was translated into Bangla for field administration and back-translated into English to ensure semantic equivalence. A pilot test was conducted to assess clarity and comprehension prior to final data collection. Part I included socio-demographic and work-related characteristics of the vendors, such as age, sex, education, monthly income, marital status, type of vending, number of items sold, working days per week, employment status, personal habits, water source, and place of residence. Part II assessed food safety practices and consisted of 23 items covering personal hygiene, workplace cleanliness, and food handling and storage. Examples included handwashing before food preparation and after toilet use, use of soap or detergent, cleaning of utensils and work surfaces, handling food while ill, use of apron or protective clothing, and reuse of cooking oil. Each item used a three-point frequency scale (always, sometimes, never). Scores were assigned as 2 for safe practice, 1 for sometimes, and 0 for unsafe practice. The total practice score ranged from 0 to 46 and was converted to a 100-point scale. Scores <50 indicated poor practice, 50-75 indicated average practice, and >75 indicated good practice [9]. Part III assessed food safety knowledge. This section evaluated awareness of food poisoning pathogens, personal and food hygiene, high-risk groups, and appropriate cleaning and handling practices. It contained 18 questions with two possible answers: yes or no. Each correct “yes” response received 1 point, and an incorrect “no” response received 0 points. Thus, the maximum attainable score was 18, which was converted to a 100-point scale. A score <50 indicated low food safety knowledge, 50-75 indicated satisfactory knowledge, and >75 was considered good knowledge [12].

Data analysis

Data were analyzed using IBM SPSS Statistics for Windows, version 27.0 (released 2010, IBM Corp., Armonk, NY). Descriptive statistics summarized the variables, and associations were examined using Chi-square tests or Fisher’s exact test. Results were presented with frequencies and percentages. A p-value <0.05 was considered statistically significant at a 95% confidence level.

Ethical consideration

Informed written consent was obtained from each participant. Ethical approval was obtained from the Institutional Review Board (IRB) of the National Institute of Preventive and Social Medicine (NIPSOM), Dhaka 1212, Bangladesh. The memo number is NIPSOM/IRB/2023/06.

## Results

Characteristics of the study participants

Table 1 shows that, among the 350 SF vendors in Rajshahi City, the majority are male (78.6%), aged between 21 and40 years (44.9%), and reside in urban areas (74.3%). Most have some level of education, with 33.7% having higher education. A large portion are business owners (74.6%) and sell multiple food items (74.0%). While many have less than five years of experience (29.1%), a significant number have five to 20 years. Income levels are modest, with most earning between 10,000 and 15,000 or more. Over half work four to six days a week (56.6%) and use supplied water (70.9%). A majority (55.7%) report no harmful habits, although some used tobacco or betel quid.

**Table 1:** Distribution of the respondents according to their sociodemographic variables (N = 350) Data presented as frequency and percentage

Name of the variables	Frequency	Percentage
Age (in years)		
20 or less	104	29.7
21 to 40	157	44.9
41 to more	89	25.4
Gender		
Male	275	78.6
Female	75	21.4
Educational qualification		
No formal education	41	11.7
Primary	107	30.6
Secondary	84	24.0
Higher	118	33.7
Working experience (in years)		
Less than 1	29	8.3
Less than 5	102	29.1
5 to 9	81	23.1
10 to 20	88	25.1
More than 20	50	14.3
Monthly income (taka)		
5000-10000	60	17.1
10001-15000	144	41.1
>15000	146	41.7
Marital status		
Single	97	27.7
Married	145	41.4
Divorced/separated	27	7.7
Widow/widower	81	23.1
Number of selling items		
One type of food item	91	26.0
More than one type of food item	259	74.0
Number of working days in a week		
Four to six days	198	56.6
Seven days	152	43.4
Employment status		
Owner	261	74.6
Employee	89	25.4
Source of water		
Supply water	248	70.9
Tube well water	71	20.3
Others	31	8.9
Personal habit		
Nothing	195	55.7
Betel quid	42	12.0
Biri/cigarette	103	29.4
Betel quid and biri/cigarette both	10	2.9
Living area		
Village	90	25.7
City	260	74.3

Compliance with food hygiene and safety practices among SF vendors in Rajshahi City

Table 2 shows that a majority reported washing hands before food processing (72.3%) and after toilet using (71.7%), a significant portion reported neglecting key hygiene steps, such as washing hands with soap (only 59.1% do so consistently) and after handling prepared food (28.3% do not). Alarmingly, 9.7% reported handling food while ill with diarrhea, and none used masks, gloves, or caps. Only 2.3% wear aprons, and 42% reported never sanitizing their working clothes. Knife and utensil sanitation was inconsistent, and many (44.6%) reported covering their mouth while coughing or sneezing. Positive practices reported by the participants include avoiding jewellery (100%) and not reusing oil (88.6%), though 40.9% still touched their face or hair during work, posing contamination risks.

**Table 2:** Distribution of the respondents by their food safety practices (N = 350) Data presented as frequency and percentage. SL: serial

SL	Food safety practices	Always frequency (%)	Sometimes frequency (%)	Never frequency (%)
1	Washing hands before food processing	253 (72.3)	0	97 (27.7)
2	Washing hands before touching unwrapped raw foods	226 (64.6)	27 (7.7)	97 (27.7)
3	Washing hands after touching unwrapped raw foods	222 (63.4)	23 (6.6)	105 (30.0)
4	Using soaps/detergents to wash hands	207 (59.1)	31 (8.9)	112 (32.0)
5	Keeping your nails short before starting activities	242 (69.1)	11 (3.1)	97 (27.7)
6	Washing hands after touching prepared foods	227 (64.9)	24 (6.9)	99 (28.3)
7	Handling food at work unhygienically while having diarrhea	34 (9.7)	0	316 (98.3)
8	Cleaning the work area before starting work	251 (71.7)	0	99 (28.3)
9	Washing hands after coming back from the toilet	251 (71.7)	0	99 (28.3)
10	Using an apron at daily work	8 (2.3)	0	342 (97.7)
11	Using a mask at daily work	0	0	350 (100.0)
12	Using a cap at daily work	0	0	350 (100.0)
13	Using gloves at daily work	0	0	350 (100.0)
14	Washing hands before using gloves	0	0	350 (100.0)
15	Washing and sanitize the clothes you use in the workplace	95 (27.1)	108 (30.9)	147 (42.0)
16	Using a tissue/cloth when coughing or sneezing	186 (53.1)	8 (2.3)	156 (44.6)
17	Washing and sanitizing the knife after chopping raw chicken or meat or other raw food	224 (64.0)	15 (4.3)	111 (31.7)
18	Using detergent to clean utensils	210 (60.0)	29 (8.3)	111 (31.7)
19	Eating and drinking in the workplace	13 (3.7)	7 (2.0)	330 (94.3)
20	Using jewelry and a wristwatch while working	0	0	350 (100.0)
21	Rubbing your hands on your face, hair, etc., while working	0	143 (40.9)	207 (59.1)
22	Smoking in the workplace	0	33 (9.4)	317 (90.6)
23	Reusing oil while preparing food	0	40 (11.4)	310 (88.6)

Knowledge of SF vendors regarding foodborne illness and consumer health risks

Table 3 identified that most respondents were aware that food poisoning can be caused by contaminated food (63.4%) and that contaminated food may lead to abortion in pregnant women, bloody diarrhea, and allergies (57.7%). Awareness declined for specific foodborne diseases, including hepatitis A (50.6%), *Salmonella* infection (45.1%), and *Staphylococcus*-related illness (42.3%). Misconceptions were evident, as 45.4% believed AIDS could be transmitted through food. Knowledge gaps were pronounced for food storage-related risks, with only 36.0% recognizing contamination from uncovered foods, overnight rice, or leftover desserts. However, 60.3% correctly understood that healthy food handlers can still transmit microorganisms.

**Table 3:** Distribution of the respondents by their knowledge regarding health hazards of consumers (N = 350) Data presented as frequency and percentage. SL: serial

SL	Knowledge regarding health hazards	YES Frequency (%)	NO Frequency (%)
1	Abortion in pregnant women can be induced by contaminated foods	202 (57.7)	148 (42.3)
2	Bloody diarrhea can be caused by contaminated foods	202 (57.7)	148 (42.3)
3	Swollen cans can contain microorganisms	202 (57.7)	148 (42.3)
4	During an infectious disease of the skin, it is necessary to take leave from work	177 (50.6)	173 (49.4)
5	Eating and drinking in the workplace increases the risk of food contamination	158 (45.1)	192 (54.9)
6	Staphylococcus is among the food-borne pathogens	148 (42.3)	202 (57.7)
7	Microbes are on the skin, nose, and mouth of healthy handlers	169 (48.3)	181 (51.7)
8	Salmonella is among the food-borne pathogens	159 (45.4)	191 (54.6)
9	Food poisoning can be caused by food	222 (63.4)	128 (36.6)
10	Influenza can be transmitted by aerosols rather than food	202 (57.7)	148 (42.3)
11	Using gloves while handling food reduces the risk of food contamination	177 (50.6)	173 (49.4)
12	Washing hands before work reduces the risk of food contamination	191 (54.6)	159 (45.4)
13	AIDS can be transmitted through food	211 (60.3)	139 (39.7)
14	Children, healthy adults, pregnant women, and older individuals are at equal risk for food poisoning	191 (54.6)	159 (45.4)
15	Food prepared in advance reduces the risk of food contamination	191 (54.6)	159 (45.4)
16	Proper cleaning and sanitization of utensils decreases the risk of food contamination	155 (44.3)	195 (55.7)
17	Reheating cooked foods can contribute to food contamination	126 (36.0)	224 (64.0)
18	Washing utensils with detergent leaves them free of contamination	126 (36.0)	224 (64.0)

Association between sociodemographic characteristics and food safety practices of SF vendors

This study found that 321 vendors (91.71%) reported poor food safety practices, while 29 (8.29%) exhibited average practices. Notably, none of the vendors met the criteria for good food safety practice. Table 4 also reveals statistically significant associations between food safety practices and several vendor characteristics. Working experience showed a strong association (χ² = 31.89, p < 0.001); vendors with less than one year of experience had poorer practices, whereas those with 10-20 years performed better. Employment status was also significant (χ² = 46.00, p < 0.001), with employees showing poorer practices than owners. Vendors working seven days a week practiced better hygiene than those working fewer days (χ² = 10.81, p = 0.001). Personal habits like chewing betel quid or smoking were significantly associated with food safety practices (χ² = 62.74, p < 0.001), with those having no such habits performing better. Living area also mattered (χ² = 10.94, p = 0.001), as city-based vendors followed better practices than village-based ones. By contrast, age (p = 0.553), gender (p = 0.074), education, and income showed no significant relationship with food safety practices.

**Table 4:** Association between vendor characteristics and food safety practices among street food vendors in Rajshahi City (N = 350) Chi-square test or Fisher’s exact test was used as appropriate based on expected cell counts. Data are presented as frequencies and percentages.

Name of the variables with categorizes	Food safety practices			
	Poor N = 321	Average N = 29	χ2	p-value
Age (in years)				
20 or less	95 (27.1)	9 (2.6)	1.18	0.553
21 to 40	142 (40.6)	15 (4.3)		
41 to more	84 (24.0)	5 (1.4)		
Gender				
Male	256 (73.1)	19 (5.4)	3.20	0.074
Female	65 (18.6)	10 (2.9)		
Educational qualification				
No formal education	38 (10.9)	3 (0.9)	2.21	0.528
Primary	101 (28.9)	6 (1.7)		
Secondary	77 (22.0)	7 (2.0)		
Higher	105 (30.0)	13 (3.7)		
Working experience (in years)				
Less than 1	20 (5.7)	9 (2.6)	31.89	<0.001
Less than 5	99 (28.3)	3 (0.9)		
5 to 9	76 (21.7)	5 (1.4)		
10 to 20	76 (21.7)	12 (3.4)		
More than 20	50 (14.3)	0 (0.0)		
Monthly Income (taka)				
5000 to 10000	60 (17.1)	0 (0.00)	6.57	0.370
10001 to 15000	130 (37.1)	14 (4.0)		
15000 to more	131 (37.4)	15 (4.3)		
Marital status				
Single	92 (26.3)	5 (1.4)	2.28	0.528
Married	132 (37.7)	13 (3.7)		
Divorced/separated	25 (7.1)	2 (0.6)		
Widow/widower	72 (20.6)	9 (2.6)		
Number of selling items				
One type of food item	83 (23.7)	8 (2.3)	0.041	0.839
More than one type of food item	238 (68.0)	21 (6.0)		
Number of working days in a week				
Four to six days	190 (54.3)	8 (2.3)	10.81	0.001
Seven days	131 (37.4)	21 (6.0)		
Employment status				
Owner	253 (72.3)	8 (2.3)	46.00	<0.001
Employee	68 (19.4)	21 (6.0)		
Source of water				
Supply water	232 (66.3)	16 (4.6)	13.28	0.001
Tube well water	58 (16.6)	13 (3.7)		
Others	31 (8.9)	0 (0)		
Personal habit				
Nothing	174 (49.7)	21 (6.0)	62.74	<0.001
Betel quid	42 (12.0)	0 (0)		
Biri/Cigarette	102 (29.1)	1 (0.3)		
Betel quid and biri/cigarette both	3 (0.9)	7 (2.0)		
Living area				
Village	90 (25.7)	0 (0)	10.94	0.001
City	231 (66.0)	29 (8.3)		

Association between vendor characteristics and knowledge of health risks related to SF consumption

This study also illustrated significant associations between vendor characteristics and their knowledge of the health risks of consumers. Gender was strongly associated (χ² = 15.03, p = 0.001), with females showing better knowledge than males. Education also had a significant impact (χ² = 22.79, p = 0.001), as those with higher or secondary education demonstrated more knowledge. Working experience (χ² = 22.10, p = 0.005) and monthly income (χ² = 26.69, p < 0.001) were both positively related to knowledge level. Vendors selling multiple food items (χ² = 13.47, p = 0.001), using supply water (χ² = 11.15, p = 0.025), and having no harmful personal habits (χ² = 46.57, p < 0.001) were more informed. Employment status also mattered (χ² = 22.45, p < 0.001), with owners being more knowledgeable than employees. However, age, marital status, and living area showed no significant association with health risks of consumers (Table 5).

**Table 5:** Association between vendor characteristics and knowledge of health hazards among street food vendors in Rajshahi City (N = 350) Chi-square test or Fisher’s exact test was used as appropriate based on expected cell counts. Data are presented as frequencies and percentages.

Name of the variables with categorizes	Knowledge regarding health hazards				
	Poor n (%)	Average n (%)	Good n (%)	χ2	p-value
Age (in years)					
20 or less	54 (15.4)	11 (3.1)	39 (11.1)	1.61	0.807
21 to 40	76 (21.7)	25 (7.1)	56 (16.0)		
41to more	43 (12.3)	12 (3.4)	34 (9.7)		
Gender					
Male	147 (42.0)	41 (11.7)	87 (24.9)	15.03	0.001
Female	26 (7.4)	7 (2.0)	42 (12.0)		
Educational qualification					
No formal education	17 (4.9)	10 (2.9)	14 (4.0)	22.79	0.001
Primary	50 (14.3)	9 (2.6)	48 (13.7)		
Secondary	39 (11.1)	6 (1.7)	39 (11.1)		
Higher	67 (19.1)	23 (6.6)	28 (8.0)		
Working experience (in years)					
Less than 1	15 (4.3)	5 (1.4)	9 (2.6)	22.10	<0.005
Less than 5	49 (14.0)	18 (5.1)	35 (10.0)		
5 to 9	49 (14.0)	1 (0.3)	31 (8.9)		
10 to 20	45 (12.9)	12 (3.4)	31 (8.9)		
More than 20	15 (4.3)	12 (3.4)	23 (6.6)		
Monthly Income (taka)					
5000 to 10000	32 (9.1)	19 (5.4)	9 (2.6)	26.69	<0.001
10001 to 15000	69 (19.7)	15 (4.3)	60 (17.1)		
15000 to more	72 (20.6)	14 (4.0)	60 (17.1)		
Marital status					
Single	48 (13.7)	16 (4.6)	33 (9.4)	10.77	0.096
Married	65 (18.6)	26 (7.4)	54 (15.4)		
Divorced/separated	13 (3.7)	1 (0.3)	13 (3.7)		
Widow/widower	47 (13.4)	5 (1.4)	29 (8.3)		
Number of selling items					
One type of food item	52 (14.9)	19 (5.4)	20 (5.7)	13.47	0.001
More than one type of food item	121 (34.6)	29 (8.3)	109 (31.1)		
Number of working days in a week					
Four to six days	93 (26.6)	29 (8.3)	76 (21.7)	1.13	0.567
Seven days	80 (22.9)	19 (5.4)	53 (15.1)		
Employment status					
Owner	120 (34.3)	48 (13.7)	93 (26.6)	22.45	<0.001
Employee	53 (15.1)	0 (0)	36 (10.3)		
Source of water					
Supply water	117 (33.4)	33 (9.4)	98 (28.0)	11.15	0.025
Tube well water	35 (10.0)	15 (4.3)	21 (6.0)		
Others	21 (6.0)	0 (0)	10 (2.9)		
Personal habit					
Nothing	83 (23.7)	33 (9.4)	79 (22.6)	46.57	<0.001
Betel quid	15 (4.3)	0 (0)	27 (7.7)		
Biri/cigarette	72 (20.6)	15 (4.3)	16 (4.6)		
Betel quid and biri/cigarette both	3 (0.9)	0 (0)	7 (2.0)		
Living area					
Village	44 (12.6)	15 (4.3)	31 (8.9)	0.969	0.616
City	129 (36.9)	33 (9.4)	98 (28.0)		

## Discussion

This study was conducted in Rajshahi City and assessed the food safety practices and health risk knowledge among SF vendors, examining their demographic characteristics, hygiene behaviors, and awareness of foodborne illness risks. Our study found that while 72.3% of vendors always washed their hands before food processing, a concerning 27.7% never do. This finding resonates with previous research conducted in Bangladesh by Rahman et al., where only 61% of vendors practiced proper handwashing before handling food [13]. Similar gaps were noted in handwashing after toilet use and after handling raw food, reinforcing the notion that consistent hand hygiene is not uniformly practiced.

The extremely low use of personal protective gear, such as aprons (2.3%), and the complete absence of gloves, caps, and masks, was particularly alarming. These findings are even lower than those reported by Muzaffar et al. in Dhaka, where only 10% of vendors used aprons, and none used gloves [14]. In a study conducted in Nigeria, Omemu and Aderoju also observed poor adherence to hygiene protocols, with only 20% using protective clothing [15], suggesting that the lack of food safety practices among street vendors is a widespread challenge in low- and middle-income countries.

Regarding food safety behavior, such as not preparing food while ill, our findings reported 9.7% working with diarrhea, which is comparable with a Kenyan study by Muinde and Kuria, where 12% of food handlers reported similar practices [5]. These behaviors significantly heighten the risk of foodborne illness outbreaks.

Our study also revealed that a significant proportion of respondents lacked knowledge about foodborne pathogens such as *Salmonella*, *Staphylococcus*, and hepatitis A. For instance, only 45.4% and 42.3% recognized Salmonella and Staphylococcus, respectively, as harmful organisms transmitted through contaminated food. These results are consistent with findings from Ethiopia by Abera et al., where only 43% of vendors knew about such pathogens [16]. However, knowledge about food poisoning was relatively higher (63.4%), reflecting partial awareness consistent with findings from a disimilar urban setup by Ma et al. in China had only 31% [12].

Misconceptions were also prevalent, such as 60.3% believing that AIDS could be transmitted via food. This echoes findings by Ma et al. in China, where 32% of food handlers held dissimilar misconceptions [12], highlighting the pervasive nature of food safety misinformation in informal sectors.

A study in Mekelle city, Northern Ethiopia, showed that food safety protocols were significantly associated with vendors' higher education levels and food safety training. About 65% had a good level of knowledge, 81.1% had a positive attitude towards food safety practices, and 58.9% had a good level of food safety practices [17].

The water source also played a pivotal role. Vendors using supply water showed better safety practices than those using tube wells or other sources. This pattern has been similarly reported in another study, where vendors with access to municipal water adhered better to hygiene practices [15,18].

Interestingly, while gender and income were significantly associated with knowledge, they were not as strongly linked to safety practices. This diverges from the findings of Barro et al., who found that income was a strong predictor of both knowledge and practice in Burkina Faso [19], suggesting that regional differences and sociocultural dynamics may mediate these relationships.

The influence of personal habits (like smoking and chewing betel quid) on both knowledge and practice (p ≤ 0.001) strongly supports findings by Choudhury et al., where similar habits were associated with poor hygiene behavior [20].

This study has several limitations. First, food safety practices were assessed using an interviewer-administered questionnaire; therefore, the findings reflect self-reported behaviors rather than directly observed practices and may be subject to social desirability and recall bias. The absence of an observational checklist or microbiological verification limits the ability to confirm actual hygiene practices and food contamination status. Second, the cross-sectional design precludes causal inference between vendor characteristics and reported food safety practices. In addition, the study did not differentiate vendors primarily involved in food preparation from those engaged mainly in serving, which may have masked role-specific differences in hygiene behavior. Finally, mobile and seasonal SF vendors were not included, which may limit the generalizability of the findings. Future studies should consider mixed-method approaches, incorporating direct observation and microbiological assessments, to validate reported practices and provide a more comprehensive assessment of SF safety.

## Conclusions

This study indicates that inadequate hygiene and limited awareness among Rajshahi’s SF vendors significantly heighten foodborne risks. Experience, education, employment, and habits influenced safety practices and knowledge. Strengthening food safety through structured training, vendor monitoring, and awareness campaigns is essential. Collaboration between local authorities and health educators should prioritize behavior change to reduce contamination and protect public health.
